# Histological study of donor/recipient feasibility in distal nerve transfer for the upper limb nerve injury

**DOI:** 10.1371/journal.pone.0322331

**Published:** 2025-05-05

**Authors:** Akira Kodama, Atsushi Kunisaki, Teruyasu Tanaka, Shigeki Ishibashi, Kentarou Tsuji, Masaru Munemori, Goki Kamei, Koji Ikegami, Nobuo Adachi

**Affiliations:** 1 Division of Regenerative Medicine for Musculoskeletal System Medical Center for Translational and Clinical Research, Hiroshima University Hospital, Hiroshima, Japan; 2 Department of Orthopaedic Surgery, Graduate School of Biomedical and Health Sciences, Hiroshima University, Hiroshima, Japan; 3 Department of Orthopaedic Surgery, Toho University School of Medicine, Tokyo, Japan; 4 Department of Anatomy and Developmental Biology, Graduate School of Biomedical and Health Sciences, Hiroshima University, Hiroshima, Japan; PGIMER: Post Graduate Institute of Medical Education and Research, INDIA

## Abstract

This study aimed to histologically investigate whether the compatibility of donor and recipient nerves in distal nerve transfer for radial and ulnar nerve palsy is suitable for restoring nerve function. Partial median to radial nerve transfer for radial nerve palsy and partial median to ulnar nerve transfer for ulnar nerve palsy were performed in 10 cadaveric upper limbs fixed using the Thiel technique. Histological analysis of the nerve samples at the coaptation site focused on the number of myelinated axons. Each recipient and donor nerve was identified in all specimens without any anatomical variations. While median-radial nerve transfer techniques showed an adequate number of donor axons, median-ulnar nerve transfer techniques showed a shortage of donor axons. The insufficiency of donor axons compared to the recipient axons may explain the challenges in reinnervating the recipient muscles. Combining the two different nerve transfers may compensate for the shortage of donor axons and improve motor recovery. Type of study and Level of evidence: Therapeutic, Level III.

## Introduction

Proximal nerve injuries in the upper limbs present a significant challenge to achieving adequate functional recovery, even with nerve repair or transplantation interventions. Historically, tendon transfer procedures such as pronator teres to the extensor carpi radialis brevis (ECRB), flexor carpi radialis (FCR) to the extensor digitorum communis (EDC), and palmaris longus to the extensor pollicis longus (EPL) for radial nerve palsy, and ECRB transfer to the adductor pollicis for ulnar nerve palsy have been the primary approaches to address these injuries [1,2]. Although these techniques have demonstrated reliability and historical success, they often result in non-physiological kinematics and reduced power grip [3]. Notably, for ulnar nerve palsy, the acquired pinch strength remains significantly below normal even after tendon transfer procedures [2,4]. Nerve transfer is a potential solution for reconstructing radial and ulnar nerve palsies [5–7]. Nerve transfers offer distinct advantages by reducing the distance from the nerve coaptation site to the target muscle compared with a nerve graft or nerve repair at the injured site. This feature enhances the effectiveness of reinnervation, reduces the required time compared to autografts, and holds the promise of restoring more physiological functions compared to tendon transfers. In radial nerve palsy, nerve transfers from the flexor digitorum superficialis (FDS) branch of the median nerve to the ECRB and from the FCR branch of the median nerve to the posterior interosseous nerve (PIN) have shown promise for minimizing the loss of recipient motor units through synergistic motor donor selection [7]. Bertelli’s observations indicated superior outcomes in wrist motion and finger extension in patients with nerve transfers compared with tendon transfers [5]. Several techniques have been reported for ulnar nerve palsy, including the distal anterior interosseous nerve (AIN) transfer to the deep branch of the ulnar nerve (DBUN) [6]. However, functional improvement has not been consistently observed in any of these reports. Some reports suggest reinnervation of the adductor pollicis (ADP) and interosseous muscles [6,8,9]. However, others have concluded that AIN-to-DBUN transfer does not provide sufficient intrinsic muscle reinnervation to prevent clawing [10,11].

Bertelli et al. introduced the concept of transferring the opponens pollicis motor branch (OPB) to the terminal division of the deep branch of the ulnar nerve (TDDBUN), which innervates both ADP and first dorsal interosseous (FDI) muscles [12]. However, clinical results remain fragmented across different institutions, lacking a consistent view of the functional prognosis and donor/recipient selection.

Critical considerations in nerve transfer surgery include factors such as the proximity of the donor nerve to the target muscle, type of axon, size match and suture tension between the donor and recipient, number of axons, and synergistic function of the original muscle compared to the recipient muscle [13]. Surgeons performing these procedures have reported inconsistent results, which may be partly due to differences in the number of axons between donor and recipient nerves [14,15]. Therefore, based on the theory that the differences in functional recovery outcomes between nerve transfer techniques may be due to the compatibility of donor and recipient nerve axon counts according to the technique used, our cadaveric study aimed to perform a histological investigation to determine how the compatibility of donor and recipient nerves varies according to the technique of distal nerve transfer for high radial and ulnar nerve palsy.

## Methods

This study was approved by the ethical review board of Hiroshima University. The approval number is E2021-2526.

### Cadaveric specimens

This study involved ten upper limbs from six cadavers donated by three females and three males aged 71–97 years (average age: 86.8 years). All cadavers were donated voluntarily by individuals during their lifetime, and consent was obtained from their families upon donation. Cadaver dissections were performed from 18 July 2021–9 July 2023. The cadavers were fixed an average of 24.7 Hours (20–28) after their death, and the cadavers were dissected an average of 166.5 days (31–353) after death. The donation and declaration forms comply with the Guidelines for Cadaver Dissection in Education and Research of Clinical Medicine in domestic societies [16] and the Act on Body Donation for Medical and Dental Education in our country. The Thiel embalming protocol, which preserves tissue flexibility and natural color, was used to preserve cadaveric specimens [17,18].

### Surgical techniques

#### Partial median nerve to radial nerve transfer for radial nerve palsy.

A volar skin incision was made over the mid-forearm, from the antecubital fossa. The distal insertion of the pronator teres is released from the radius to facilitate exposure of the median nerve. After identification of the radial nerve, the PIN was tracked distally and released from the supinator. The superator branches were separated from the PIN.

The FCR branch of the median nerve is transferred to the PIN (FCR/PIN), and the FDS branch to the ECRB (FDS/ECRB). When multiple FDS branches were present, the branch with the largest diameter was selected as the donor nerve ().

#### Partial median nerve to ulnar nerve transfer for ulnar nerve palsy.

The following techniques were performed in anticipation of nerve transfer in ulnar nerve palsy. The terminal branch of the AIN was transferred to the DBUN (AIN/DBUN) as previously described [19].

The incision was made from the level of the Guyon Canal to the mid-forearm. The DBUN was identified within the Guyon Canal and dissected between the DBUN and the superficial branch of the ulnar nerve(SBUN). The AIN was cut where it started to branch in the middle portion of the pronator quadratus and mobilized proximally so that it could be transposed to the ulnar nerve (Fig 2). The DBUN was cut at the coaptation site and the distal cut end was brought into contact with the AIN for coaptation.

**Fig 1:**
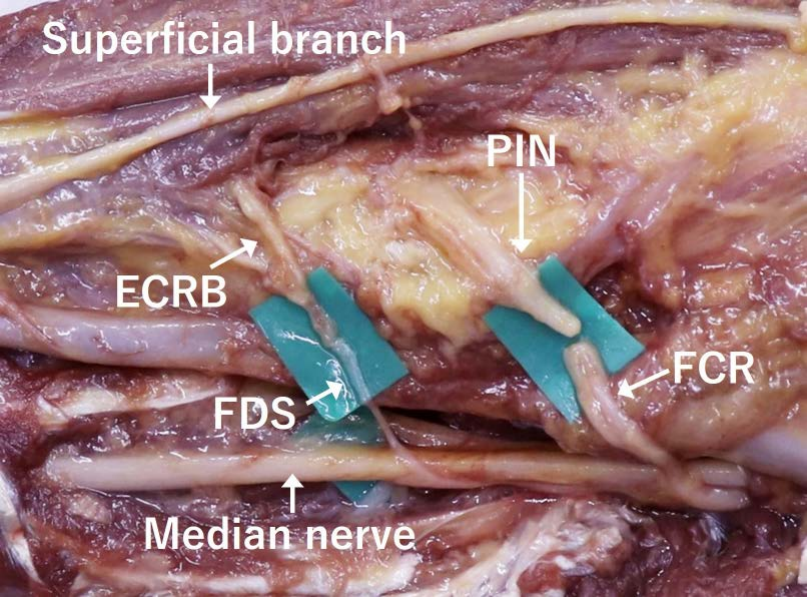
Transfer of the flexor carpi radialis (FCR) to the posterior interosseous nerve (PIN) and the flexor digitorum superficialis (FDS) to the extensor carpi radialis brevis (ECRB) at the proximal forearm.

In addition, we performed a technique that transferred OPB to TDDBUN [12]. A skin incision was made over the first web space through the thenar region and down to the carpal tunnel. The recurrent branch of the median nerve was dissected from the carpal tunnel. The OPB was identified by its location deep in the abductor pollicis brevis (APB) muscle and was dissected intramuscularly. We identified TDDBUN by identifying the ADP muscle and separating the adductor heads deep into it (Fig 3).

**Fig 2:**
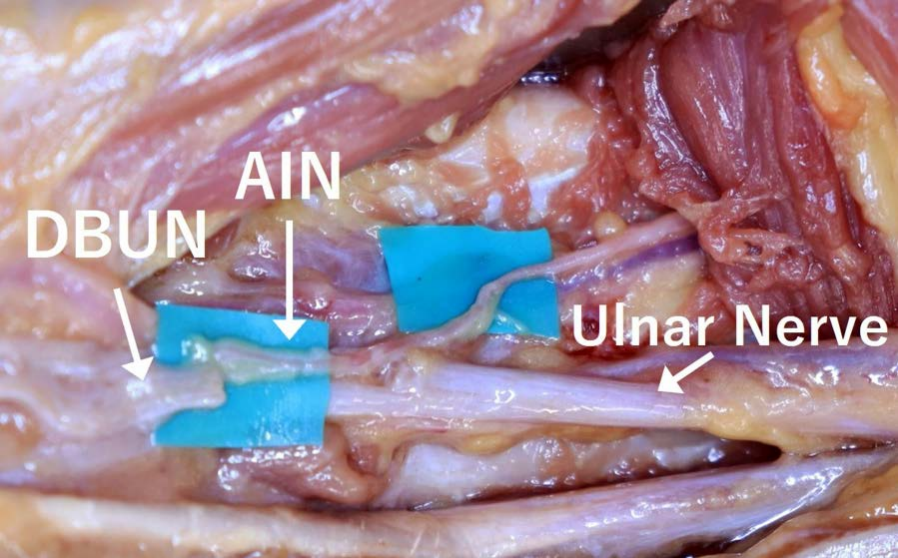
Transfer of the AIN to the DBUN at the distal forearm.

**Fig 3:**
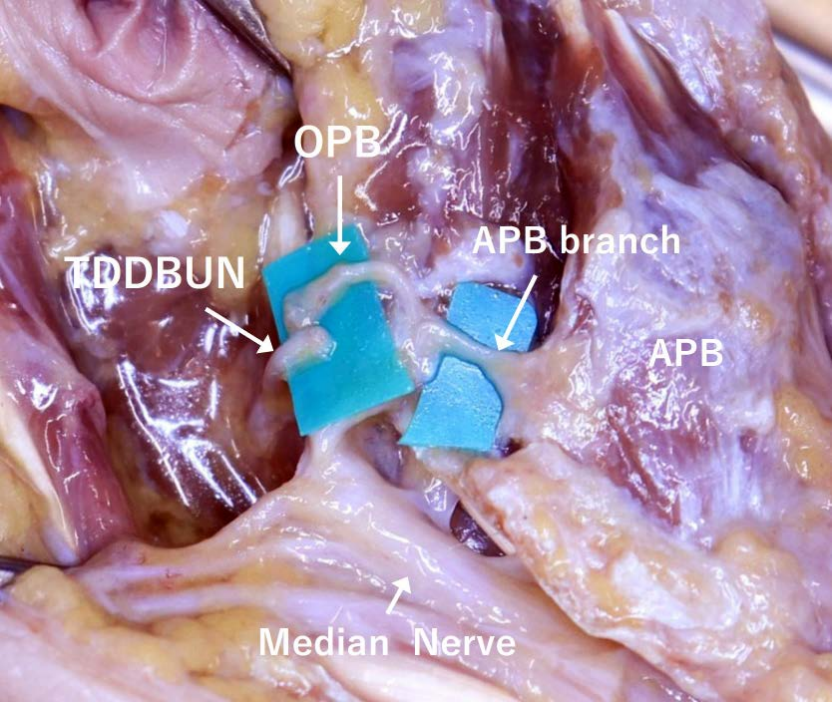
Transfer of the OPB to the TDDBUN at the thenar.

In each technique, the donor and recipient nerves are moved to the coaptation site. After confirming that suturing was possible, the nerve was harvested at 10 mm from the coaptation site.

### Histological analysis

Nerve samples were collected from each specimen at the level of the coaptation site and then fixed at 4 °C in 2.5% glutaraldehyde (Nacalai Tesque Inc., Kyoto, Japan) with 0.1 M Millonig’s buffer (pH 7.4) for 2–8 weeks. After post-fixation in 1% aqueous osmium tetroxide, samples were dehydrated in an ascending series of alcohols (50–100%) and propylene oxide (Katayama Chemical, Osaka, Japan). The samples were embedded in an epoxy resin (Nisshin EM Co. Ltd., Tokyo, Japan) to preserve their orientation, and cured at 60 °C for 48 h. Multiple 1 μm semithin transverse sections were cut with an ultramicrotome (Reichert-Jung, Leica, Tokyo, Japan) and stained with 1% toluidine blue (Sigma-Aldrich, St. Louis, MO, USA) for 1 min.

Photomicrographs of these sections were obtained using a fluorescence microscope (KEYENCE, Osaka, Japan) connected to a digital camera and computer. The nerve diameter, fascicle number, and cross-sectional area of the individual fascicles were measured at 100X magnification, followed by the number of myelinated axons at 200X magnification. The cross-sectional areas were measured using ImageJ software (National Institutes of Health, Bethesda, MD, USA). The total fascicle area was calculated as the sum of the cross-sectional surfaces of all the fascicles. Myelinated axons were counted using ImageJ software. The cutoff value for the inclusion of axons was 4 μm. Axon density was calculated as the ratio of the axon number to the fascicle area. The donor-to-target ratios of the axon numbers were also calculated.

### Statistical analysis

The donor and recipient nerve values for each nerve transfer technique were compared using Student’s t-test, with p ≤ 0.05 considered significant. All data are expressed as the mean ± standard deviation (SD), with appropriate ranges.

## Results

Each recipient and donor nerve was identified in all specimens, without anatomical variations. Furthermore, in each specimen, all donor nerves could contact the recipient nerves without tension.

**Fig 4:**
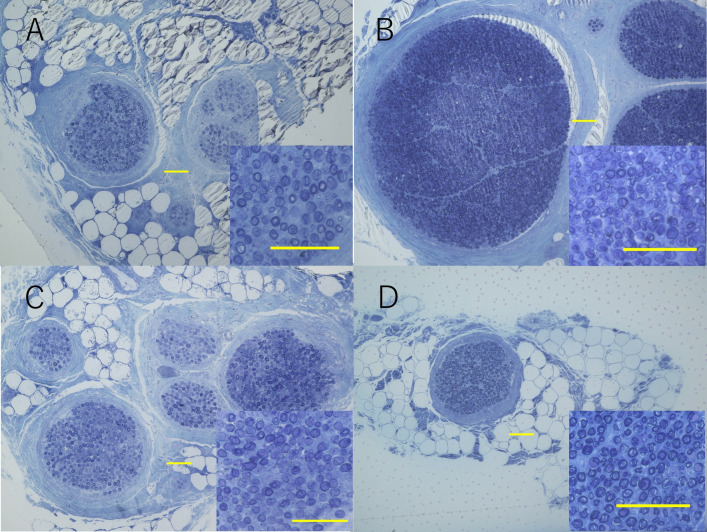
Semithin sections of the FCR (A), PIN (B), FDS (C), and ECRB (D) from the coaptation site. Calibration bars represent 100 µm.

For the partial median nerve to radial nerve transfer, a comparison of the donor and recipient nerves revealed that the FCR had a significantly smaller fascicle area and a significantly lower number of axons than the PIN. In contrast, FDS had a similar fascicle area and number of axons as ECRB, showing no significant differences (Table 1, Fig 4).

**Fig 5:**
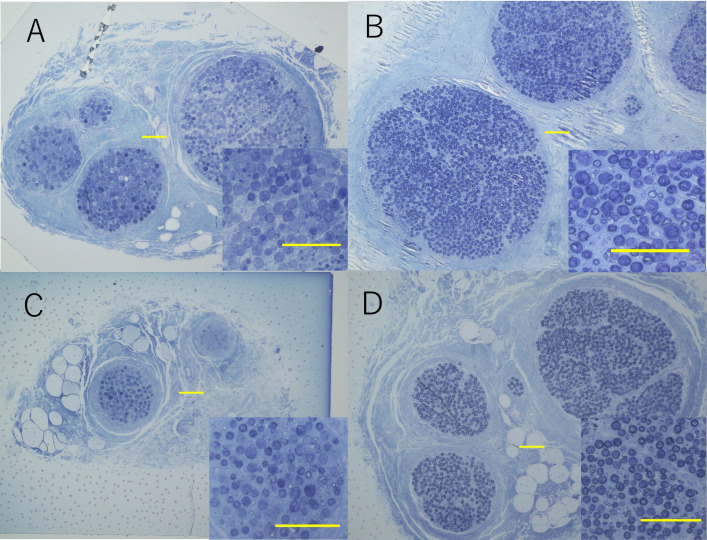
Semithin sections of the AIN (A), DBUN (B), OPB (C), and TDDBUN (D) from the coaptation site. Calibration bars represent 100 µm.

In the partial median nerve to ulnar nerve transfer, the AIN had a significantly smaller fascicle area and fewer fascicles and axons than the DBUN. OPB was also inferior to TDDBUN in terms of fascicle number, fascicle area, and axon number (Table 2, Fig 5). The individual donor-to-recipient axon number ratios for each technique were as follows: FCR/PIN, 1:3.8; FDS/ECRB, 1:0.9; AIN/DBUN, 1:5.2; OPB/TDDBUN, 1:4.5. Axon density was not significantly different between the donor and recipient for any nerve transfer technique.

**Table 2:** Histological findings of the median to the ulnar nerve transfer.

	Number of fascicles^††^	Fascicle Area ^†^(mm^2^)	Axon Density^†^ (axons/mm^2^)	Axon Number^†^
AIN	3.6(1-7)	0.27(0.07)	3623(716)	951(179)
DBUN	7.6(5-10)	1.21(0.36) **	4196(931)	4961(1511) **
Donor/Recipient		1:4.5	1:1.2	1:5.2
OPB	1.2(1-2)	0.09(0.02)	3597(531)	316(124)
TDDBUN	3.1(1-6)	0.45(0.22) **	3325(625)	1413(478) **
Donor/Recipient		1:5.3	1:0.9	1:4.5

Data are expressed as mean (SD)^†^ and mean (range)^††^.

*p<0.05, **p<0.01, Student’s t-test.

## Discussion

Of the four types of nerve transfers examined in this study, all methods other than FDS/ECRB resulted in a significantly lower number of donor nerve axons than the recipient. However, these techniques have been reported to restore useful functions in clinical studies [5–8,12,19,20].

Thus, nerve transfer using a donor nerve with fewer axons than that of the recipient nerve may be successful. An animal study using rat tibial nerves showed that one axon in the proximal stump could maintain up to three or four collateral sprouts [21]. Animal experiments on nerve transfer with different donor-to-recipient axon ratios using rabbit upper extremity models have shown that 50% of the normal muscle power is achieved after one-third ulnar nerve transfer to the median nerve. However, the number of axons has not been examined [22].

Given these findings, we assumed that a lack of donor axons could be tolerated up to a donor-to-recipient axon number ratio of 1:3, and compared the donor/recipient suitability for each technique. The donor/recipient axon count ratios for each procedure in the previous cadaver study are shown in Table 3.

**Table 3:** Donor to recipient ratio of the axon number compared with previous reports.

	Axon number		Donor/Recipient Ratio
	Donor	Recipient	
	FCR	PIN	
Current study	1414	5350	1:3.8
Sukegawa (2016)	1501	5162	1:3.4
Martin(2022)	1223	5872	1:5
Cheah(2019)	746	3031	1:4
	FDS	ECRB	
Current study	874	763	1:0.9
Sukegawa (2016)	885	548	1:0.6
Cheah (2019)	883	745	1:0.8
	PT	ECRL	
Martin(2022)	858	790	1:0.9
Cheah(2019)	625	704	1:1.1
	AIN	DBUN	
Current study	951	4961	1:5.2
Schenck (2014)	600	2900	1:4.8
Üstün (2001)	912	1216	1:1.3
	AIN	DBUN-TDDBUN^†^	
Current study	953	3548	1:3.7
	OPB	TDDBUN	
Current study	316	1413	1:4.5
Bertelli 2019	392	619	1:1.6

† Number of axons in recipient with AIN as donor when AIN/DBUN and OPB/TDDBUN transfer are performed simultaneously.

In the FCR/PIN and FDS/ECRB transfers, both our results and those of a previous study [23] showed that a donor-recipient ratio of 1:3 was almost met, suggesting that a sufficient number of donor axons were obtained in the median to radial nerve transfer. On the other hand, another Cadavor study chose PT/ECRL for wrist extension reconstruction, with a donor-recipient ratio of 1:0.9, comparable to our FDS/ECRB results.PT/ECRL is also a promising method to restore function [24]. However, PT-ECRL is often used for tendon transfer as an aid for wrist extension until nerve recovery is achieved when performing nerve transfer with FDS/ECRB [3,7]. Martin’s study states that the FCR/PIN lacks the donor axons needed to restore function. The difference from our results may be due to the fact that FCR have individual differences in the number of branches, which may lead to variations in the number of axons in each Branch. Cheah et al. harvested the distal ends of the primary nerve branches of 23 upper limb muscles from 10 fresh-frozen cadaveric upper limbs for quantitative histomorphometry [25]. Their results were similar to ours in terms of Median to Radial nerve transfer. However, this study sampled the distal end of the primary nerve branch of the muscle, which is a different harvest site from the nerve coaptation site that simulates nerve transfer (Table 3).

In contrast, in median-ulnar nerve transfers, donor nerves were significantly deficient in AIN/DBUN, with a donor/recipient ratio of 1:4.8 in this study and in a study by Schenck et al. [15]. Üstün et al. reported a higher rate of donor axons compared with our results [26]. Still, this difference may be because the samples were taken from the level of the pisiform, which is not the original coaptation site, as Schenck et al. mentioned [15]. Although the SBUN and DBUN were identified at the level of the Guyon’s canal, and these nerves were divided proximally, crossing fascicles were observed between the two branches, and differences in technique in deciding whether to include these in the DBUN or SBUN may have resulted in different axon counts in the recipients.

For OPB/TDDBUN transfer, although the donor/recipient ratio values differed between our results and those reported by Bertelli et al.[12], the number of donor axons is considered to support functional recovery. The cause of this discrepancy in the results remains uncertain; however, the difference between our measurements and those in the literature can be partially explained by the inclusion and exclusion criteria in axon identification. However, because there is only one previous report, further verification is required.

These results suggest that the number of donor axons in median-to-radial nerve transfer is adequate. However, the number of donor axons in median-to-ulnar nerve transfer is inadequate, especially for AIN/DBUN. A previous anatomical study suggested that insufficient FDI and ADP reinnervation after nerve transfer from the AIN to the DBUN may be due to insufficient axons in the AIN compared with the DBUN [15]. Some studies have stated that this inadequate reinnervation may be due to the low donor-to-recipient axon ratio and insufficient axons in AIN [10,11].

Combining AIN-DBUN and OPB-TDDBUN can compensate for the shortage of donor axons in the AIN/DBUN. In addition, the TDDBUN component of the DBUN can be subtracted from the number of recipient axons donated by the AIN, thus compensating for the lack of donor axons in AIN-DBUN transplantation. Indeed, the donor-recipient AIN:(DBUN-TDDBUN) ratio improved to 1:3.5 when AIN-DBUN and OPB-TDDBUN were performed simultaneously. (Table 3) Based on the results of this study, we recommend the combined nerve transfer of AIN-DBUN and OPP-TDDBUN for ulnar nerve palsy. As the two surgical sites are separated into the forearm and palm, simultaneous surgeries do not affect each other.

Several studies have histologically tested the compatibility of donor recipients with upper-limb nerve transfers using cadavers [15,23,24,26]. However, each of these studies examined only one technique. The strength of our study is that, in addition to verifying the results of existing studies, it clarifies the differences in the donor-recipient ratio for each nerve transfer technique by evaluating several techniques in the same cadaver. Evaluating multiple techniques can help predict functional outcomes between procedures and provide simulation guidance, such as the simultaneous use of the AIN-DBUN and OPB-TDDBUN for ulnar nerve palsy, as demonstrated in this study.

However, a donor/recipient ratio of at least 1:3 was preferable in our study. This idea was based on the findings of Jiang et al., who reported that up to 3–4 collaterals can be developed by one axon. In contrast, functional compensation is often observed in partially denervated muscles [27]. Gordon et al. described that single motor units enlarged to approximately five times their original size, resulting in the ability to compensate for up to 80% of motoneuron loss [28]. Based on motor unit enlargement, nerve coaptations with an axon ratio between 1:3 and 1:5 can be expected to have the same motor recovery ability as that of coaptations with an optimal ratio greater than 1:3.

This study has some limitations. First, the specimens used in this study were fixed cadavers rather than living organisms. Samples from fixed cadavers may differ in thickness from the nerve bundles and myelin sheaths in the live body. However, there is no difference in the number of axons between living and fixed tissues.

Second, the average age of the cadavers used in this study was 86.8 years old, which is much higher than the age range of patients who actually require nerve transfer surgery. A reduction in the diameter or density of myelinated fibers has been reported with aging in the peripheral nerves of humans and several animals [29,30]. The relationship between age and number of axons is unclear, although it is possible that they decrease. However, it is possible to compare the donor and recipient nerve sizes within the same individual. In addition, the surgical techniques used in the cadaver study influenced the results of the study, such as a larger surgical field than in actual surgery, making the surgery easier to perform and overlooking vascular preservation and hemostasis. Finally, soft tissue flexibility was reduced in fixed cadavers compared with living or freshly frozen cadavers, which may have affected the distance and route of nerve transfer. However, because Thiel embalming can retain flexibility close to that of actual living tissue, it has been used for several types of surgical training [31,32], 3D anatomical studies of the brachial plexus [33], and anatomical studies to avoid proximal radius PIN injuries during posterior and lateral approaches [34]. The simulation of nerve transfer in this study allowed for the same surgical technique as in living bodies, and the impact of thiel embalming on the surgical technique was considered limited.

**Table 1:** Histological findings of the median to the radial nerve transfer.

	Number of fascicles^††^	Fascicle Area^†^(mm^2^)	Axon Density^†^ (axons/mm^2^)	Axon Number^†^
FCR	4.4(1-7)	0.29(0.10)	4553(994)	1414(578)
PIN	3.7(1-7)	1.11(0.32) **	4988(1061)	5350(1488) **
Donor/Recipient		1:3.8	1:1.1	1:3.8
FDS	4.1(2-8)	0.19(0.11)	4717(642)	874(443)
ECRB	2.1(1-3)	0.17(0.07)	4419(474)	763(307)
Donor/Recipient		1:0.9	1:0.9	1:0.9

Data are expressed as mean (SD)^†^ and mean (range)^††^.

*p<0.05, **p<0.01, Student’s t-test.
